# Genetic Diversity in HIV-1 Subtype C LTR from Brazil and Mozambique Generates New Transcription Factor-Binding Sites

**DOI:** 10.3390/v6062495

**Published:** 2014-06-23

**Authors:** José Boullosa, Mahesh Bachu, Dulce Bila, Udaykumar Ranga, Theodoro Süffert, Tomoko Sasazawa, Amilcar Tanuri

**Affiliations:** 1 Department of Genetics, Universidade Federal do Rio de Janeiro, Avenida Carlos Chagas Filho, 373. Edifício do Centro de Ciências da Saúde, Bloco A, sala A2-121, Cidade Universitária, Cep: 21.941-902, Brazil; E-Mails: joseneto@biologia.ufrj.br (J.B.); dulcebila@gmail.com (D.B.); 2 Jawaharlal Nehru Centre for Advanced Scientific Research, 560 064, Jakkur Post, Bangalore, India; E-Mails: bmahesh07@gmail.com (M.B.); udaykumar@jncasr.ac.in (U.R.); 3 Prefeitura Municipal de Porto Alegre, Rio Grande do Sul, 90010-170, Brazil; E-Mail: tasuffert@terra.com.br; 4 Prefeitura Municipal de Curitiba, Paraná; 80000-000, Brazil; E-Mail: tito@sms.curitiba.pr.gov.br

**Keywords:** HIV-1, Subtype C, LTR, insertion, NFkB, RBEIII, Brazil, Rio Grande do Sul, Paraná, Mozambique, MFNLP

## Abstract

The HIV-1 subtype C has been substituting the subtype B population in southern Brazil. This phenomenon has been previously described in other countries, suggesting that subtype C may possess greater fitness than other subtypes. The HIV-1 long-terminal repeat (LTR) is an important regulatory region critical for the viral life cycle. Sequence insertions immediately upstream of the viral enhancer are known as the most frequent naturally occurring length polimorphisms (MFNLP). Previous reports demonstrated that the MFNLP could lead to the duplication of transcription factor binding sites (TFBS) enhancing the activity of the HIV-1 subtype C LTR. Here, we amplified and sequenced the LTR obtained from proviral DNA samples collected from patients infected with subtype C from the Southern Region of Brazil (naïve or treatment failure) and Mozambique (only naïve). We confirm the presence of different types of insertions in the LTR sequences of both the countries leading to the creation of additional TFBS. In the Brazilian clinical samples, the frequency of the sequence insertion was significantly higher in subjects experiencing treatment failure than in antiretroviral naïve patients.

## 1. Introduction

Human Immunodeficiency Virus type 1 is the main causative agent of the AIDS pandemic. The virus evolved quickly, and generated 9 genetic related subtypes as well 52 circulating recombinant forms (CRF). Among all these variants, Subtype C strains are responsible for more than half of infections worldwide [1]. In some areas such as in southern Brazil subtype B HIV-1 epidemic has been replaced by subtype C and this variant is now accountable for more than 75% of the new infections [2]. Thus far, the impact of the huge genetic variation on the biological and pathogenic properties of HIV-1 is not well understood. Experimental evidence, however, is suggestive of greater mucosal transmission [3] of subtype C as well as faster accumulation of drug resistance mutations in subtype C isolates [4].

Subtype-specific molecular variations can modify viral regulatory sequences leading to different responses of the cis/acting regulatory elements. Some of these molecular variations have been mapped to the viral promoter where host and viral transcription factors bind to regulate viral transcription. HIV-1 promoter located at the 5' long terminal repeat (LTR) region contains several TFBS. In the viral promoter, subtype-associated genetic differences are noticeable in TFBS including the nuclear factor kappa B (NF-κB), the nuclear factor of activated T cells (NFAT) and the upstream stimulating factor (USF) elements, and in other regulatory elements such as the TATA box, and the trans-activation response element region (TAR). Among the HIV-1 subtypes, the subtype C LTR represents the most genetically divergent sequence [5]. The insertions in the LTR immediately upstream of the viral enhancer are known as the most frequent naturally occurring length polymorphism (MFNLP), and these features are well described in the context of HIV-1 subtype B [6]. The most significant genetic variation associated with subtype C is the presence of 3 NF-κB motifs in the LTR [7]. In contrast, in most of the other viral subtypes including subtype B only two genetically identical NF-κB motifs are present [8]. NF-κB is one of the key nuclear transcription factors involved in the initial steps of HIV-1 transcription trigging the viral replication through TAT/TAR mediation. Variation in LTR was also observed in CRF1 where NF-κB site has been replaced by a GA binding protein (GABp) motif [8].

Although a large proportion of the subtype C LTRs contains three NF-κB sites, a recent study of subtype C LTR isolates (n = 25) of India, found a significant proportion of subtype C LTRs characterized by the presence of a fourth NF-κB motif or an NF-κB like site [9]. In this publication, the authors reported at least three different types of sequence insertions. Nearly half of the viral isolates examined contained as additional NF-κB site (12/25), while others contained an NF-κB-like site (4/25) or an additional RBEIII (6/25) motif. Interestingly, in the follow up report, the authors observed that individuals infected with the subtype C isolates carrying four NF-κB motifs could exhibit a higher viral load as compared to those carrying the viral isolates containing only 3 NF-κB motifs. Of note, the two groups could not be distinguished from each other with respect to the further, a functional evaluation of the fourth NF-κB motif using gel shift assays confirmed the binding of NF-κB to this sequence and enhancing the transcription activity of the viral promoter as compared to the counterpart carrying only three such motifs [10]. In Brazil, the presence of HIV-1 subtype C samples containing three NF-κB binding sites in LTR was previously described [11].

There are some reports supporting the hypothesis that differential distribution of HIV-1 subtypes in the human population may reflect differences in the replication fitness and the transmission efficiency of different subtypes and recombinant forms [12,13,14]. The viral fitness represents the ability to adapt to an environment. In viruses, the concept relates to the replicative capacity of different viral quasispecies in an organism exposed to different selective pressures. An association between *ex vivo* fitness and progression to AIDS was previously shown [15]. In extracellular environment, the interaction between envelope proteins and the receptors and co-receptors affects the viral fitness [16]. However, in the intracellular environment, the viral promoter could be an important factor determining the viral fitness. The HIV-1 LTR contains different binding sites for an array of cellular transcription factors in addition to having a binding site for the Tat protein evolved in initiation and the elongation of transcription thus impacting the viral replicative capacity [17]. Of note, Bachu *et al*. showed that the sequence insertions in LTR region of HIV-1 subtype C could generate a new site for the binding of NF-κB, and these variant viral strains showed an enhanced replicative capacity [10].

In Brazil, HIV-1 subtype B is the most prevalent viral subtype. In the Southern Region, that includes Rio Grande do Sul and Santa Catarina, the prevalence of the subtype C, however, is on the rise [18]. The population change in countries where subtype C is introduced is a characteristic phenomenon of this HIV-1 variant. This was clearly noticed in some sub-Saharan African countries such as South Africa and Tanzania [19,20] and in India [21].

In this work, we characterized the genetic variation in HIV-1 LTR from subtype C isolates derived from the southern region of Brazil and Mozambique in order to describe the transcriptional factor motifs present in these isolates, with special interest in the new NF-κB previously reported by Bachu *et al*. [9] in Indian samples.

## 2. Results and Discussion

Fifty samples from Maputo, Mozambique and, 65 from Paraná and Rio Grande do Sul, Brazil were selected based on the subtype assignment in the pol region. The samples were screened using primers reported by Siddappa *et al.* [22] to obtain an amplicon of ~160 base pairs containing the core of the LTR regulatory sequences (HXB2 coordinates 256 to 393). The PCR products were subjected to agarose gel electroforesis to identify the viral isolates containing insertions. Overall, we found 48 samples containing putative insertions in the core domain of the LTR.

The prevalence of the sequence insertion in our samples was variable between the two groups tested. In the drug naive group derived from Rio Grande do Sul and Paraná, 57% (12 of 21) and 36% (5 of 14) of the viral isolates contained a sequence insertion, respectively. In contrast, in the group of patients failing ARV treatment, samples from Rio Grande do Sul and Paraná contained a sequence insertion at a prevalence of 63% (10 of 16) and 50% (7 of 14), respectively. In Mozambique 12 of the 50 viral isolates (24%) examined showed a presence of the insertions in the LTR. Although a trend toward association between antiretroviral therapy and the presence of insertions can be observed, this association did not reach a statistically significant difference (*p* > 0.05).

The near full-length LTR (~512 bp) of 31 of 115 viral isolates could be successfully determined using the nested PCR reported previously [10]. The second-round PCR products were sequenced on both the strands, the sequences aligned using Mega 5 software [23] and the important regulatory motifs were identified. All the Brazilian and the Mozambican LTR sequences containing insertions were further genotyped using the LTR subtype reference sequences downloaded from the Los Alamos Laboratory (Los Alamos, CA, USA) sequence database. The phylogenetic analysis confirmed that, in concordance with pol region, most of sequences clustered with subtype C. However, we found two putative mosaic samples, both derived from Paraná, Brazil, that clustered in LTR with subtype B (sample 18) and subtype D (sample 19) although they were subtype C in pol and RT(see Table 1 and Supplementary Figures S1–S3).

**Figure 1:**
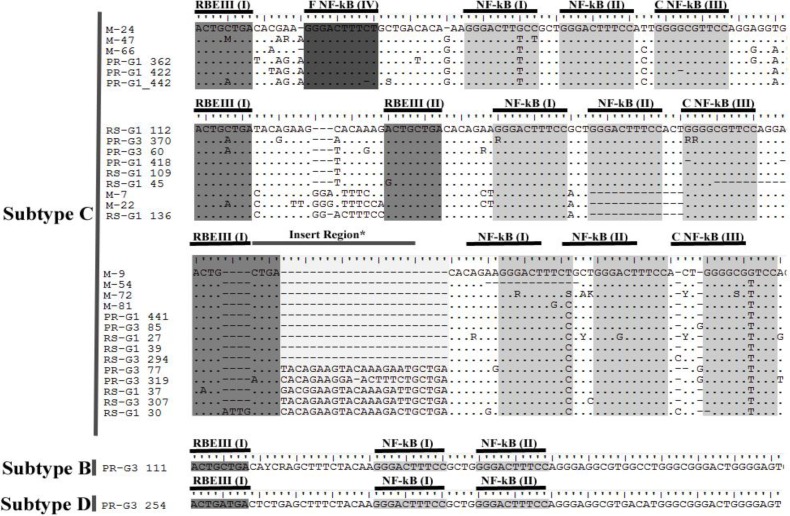
Profile of the sequence insertion in the long-terminal repeat (LTR) region from Brazil (PR and RS) and Mozambique (M).

After manual editing, the sequences were aligned and the insertions in the LTR region were categorized (Figure 1). Among the samples showing inserts in LTR, four distinct types of insertion were identified. In the first group, three isolates from Mozambique and three from Parana, Brazil from drug naïve group showed a fourth NF-κB binding site, similar to those found in the Indian viral isolates [9]. Nine isolates showed an additional RBEIII site embedded within a polymorphic 21 nucleotide (nt) insertion. Additionally we observed another group of specimens with similar NF-κB sites having differences of up to two nucleotides relative to the canonical NF-κB binding site (M-7, M-22, RS-G1 136 and PR-G3 319). Interestingly, three of these samples presented a deletion in one of the typical NF-κB site; maybe the new site has compensatory function for this loss. A fourth category of specimens was found carrying a long stretch of sequence insertion with no known new TFBS. Further, as described above, two of the viral isolates appeared to be mosaic viruses with the LTR in these cases belonging to subtype B or D.

The focus of the present study was to report the occurrence of insertions in the LTR of HIV-1 subtype C in samples derived from southern Brazil and Mozambique and compare the nature of the sequence insertion with that of India reported previously. Initially our aim was to try to check the presence of a fourth functional NF-κB. Of note, in India, subtype C isolates seems to be very polymorphic since there is approximately 6% of genetic distance across LTR region analyzed [9]. The Mozambican samples showed genetic distance score of 7.5% while the Brazilian samples appear to be closer, with a score of 3.3%. Overall, we observed a significant difference in the frequency of the insertion found in Indian samples that created a new NF-κB site between Brazil and Mozambique. In fact, in Mozambique the presence of this insertion generated the fourth NF-κB site (3 in 5; 60%) while in Brazil we could only found 3 in 14 analyzed (21%). The majority of insertion found in Brazilian samples generated a new RBE III site. We should note that the presence of subtype C in Africa is prior to the entry of this subtype in Brazil, moreover, its presence in Brazil is concentrated in Southern states like Rio Grande do Sul, Santa Catarina and Parana showing a gradient of prevalence of this variant. In fact, subtype C is the major subtype found in the Rio Grande do Sul and Santa Catarina accounting for more than 75% of new infections since 2010 and Parana shows a prevalence of 25% of this variant in newly infected individuals [18]. Remarkably, we only found these insertions generating the fourth NF-κB site in samples from Parana in drug naïve group. Parana is a state with a new subtype C epidemic [24]. In India, these insertions showed a time-related growth where, in surveys targeting recent infected individuals, the prevalence of these insertions is higher.

**Table 1:** The clinical profile and the sequence details of the samples analyzed in this study.

*Sample n^o^*	*Sample Code*	*Subtype*			*CD4 (cell/mm^3^)*	*Viral Load (copies/mL)*	*Presence of MFNLP*		
		*LTR*	*PR*	*RT*			*NF-κB F*	*NF-κB like*	*RBE-III*
1	M-7	C	-	C	314	61.252	−	+	+
2	M-9	C	-	C	731	3.312	−	−	−
3	M-22	C	-	C	303	12.745	−	+	+
4	M-24	C	-	C	339	27.756	+	−	−
5	M-47	C	-	C	235	11.246	+	−	−
6	M-54	C	-	C	231	92.079	−	−	−
7	M-66	C	-	C	574	2.982	+	−	−
8	M-72	C	-	C	561	490.032	−	−	−
9	M-81	C	-	C	554	6.796	−	−	−
10	PR-G1 362	C	C	C	765	156	+	−	−
11	PR-G1 418	C	C	C	815	105.948	−	−	+
12	PR-G1 422	C	C	C	523	36.696	+	−	−
13	PR-G1 441	C	C	C	523	17.279	−	−	−
14	PR-G1 442	C	C	C	637	3.772	+	−	−
15	PR-G3 60	C	C	C	148	-	−	−	+
16	PR-G3 77	C	C	C	-	-	−	−	−
17	PR-G3 85	C	C	C	89	10	−	−	−
18	PR-G3 111	**B**	C	C	218	29.728	−	−	−
19	PR-G3 254	**D**	C	C	-	-	−	−	−
20	PR-G3 319	C	C	C	80	340.000	−	−	−
21	PR-G3 370	C	C	C	28	-	−	−	+
22	RS-G1 27	C	C	C	-	-	−	−	−
23	RS-G1 30	C	C	C	-	-	−	−	−
24	RS-G1 37	C	C	C	15,406	842	−	−	−
25	RS-G1 39	C	C	C	438	8.234	−	−	−
26	RS-G1 45	C	C	C	655	564	−	−	+
27	RS-G1 109	C	C	C	721	656	−	−	+
28	RS-G1 112	C	C	C	1356	140	−	−	+
29	RS-G1 136	C	C	C	877	9.284	−	−	+
30	RS-G3 294	C	C	C	190	249	−	−	−
31	RS-G3 307	C	C	C	-	-	−	−	−

## 3. Experimental Section

### 3.1. Samples

The Brazilian samples were collected from patients followed by the DST/Aids program from Brazilian Ministry of Health (Brasilia, Brazil). All patients are resident on the states of Parana (Curitiba, Brazil) and Rio Grande do Sul (Porto Alegre, Brazil) and were divided in two groups based on virological status, the drug naïve group was called Group I (GI) and the patients in therapeutic failure is Group II (GII). All the patients are followed by the Brazilian HIV/AIDS program, and the patients in the Group II were considered adherent to the antiretroviral therapy by drug pick-up report by the clinics. The patients was considered to be in therapeutic failure whenever they showed, on two occasions, a viral load above 50 copies/mL after 24 weeks of treatment [25]. All the samples were previously genotyped as subtype C in reverse transcriptase and protease using REGA Subtyping Tool [26,27]. The Mozambican samples were collected at Centro de Saúde do Alto Maé (Maputo, Mozambique) and all of them are genotyped as subtype C based on RT sequence (Table 1).

Whole blood sample was collected from each patient, and a portion was used to CD4 count and viral load. The reminiscent material was used to buffy coat separation and gDNA extraction using the QIAamp MiniElute DNA Kit (Qiagen, Valencia, CA, USA) according to manual. The DNA samples were quantified and stored at −20 °C.

All subjects were informed and signed the consent under the approval of the bioethical and academic committees in both countries.

### 3.2. PCR Amplification

The insertions were identified by nested PCR using the primers N419F (5' GAT GGT GCT TCA AGC TAG TRC CAG TTG A 3') e N424R (5' CTC TAT YTT RTC TAA RGC TTC YTT GGT GTC 3') on the first round and N415F (5' AGT GGA AGT TTG ACA GTC AMC TAG CAC RC 3') e N417F (5' CGC CCA GAC CAC WCC TCC TGR AMC GC 3') on the second round. The PCR conditions were as follows: In the first round, 50 ng of gDNA were added to the reaction with 1× reaction buffer (Invitrogen, Carlsbad, CA, USA), 3 mM MgCl_2_, 200 µM each deoxinucleotide, 25 pMole each primer and 0.625 U *Taq* Platinum DNA Polimerase (Invitrogen), in a final volume of 25 µL. The PCR conditions were: 94 °C (1 minute), 60 °C (1 minute) and 72 °C (1 minute) in 35 cycles total. On the second round, 2 µL from the first round were added to the reaction. The PCR conditions were: 94 °C (1 minute), 60 °C (1 minute) and 72 °C (40 seconds) in 35 cycles total. The products were submitted to 2% agarose gel electrophoresis and visualized using ethidium bromide under UV light.

The positive samples were submitted to a new nested PCR to amplify near total LTR using the primers N558 (5' TGG AAG GGT TAA TTT ACT CTA AGG AAA GGA AAG AGA TCC TTG 3') and N424 (5' GAC ACC AAR GAA GCY TTA GAY AAR ATA GAG 3') on the first round and N698 (5' ATG ACG ACG CGT TGG AAG GGT TAA TTT ACT CYM AGA AAA GRC AAG A 3') and N854 (5' GAA TTC CTG CTA GAG ATT TTC CAC ACT ACC AAA AG 3') on the second round. The products were submitted to a 2% agarose gel electrophoresis to confirm the size.

### 3.3. Sequencing

Near full length LTR fragments were sequenced in a 3100 ABI Prism (Applied Biosystems, Grand Island, NY, USA) using the Big Dye Terminator Sequencing Kit according to the manual. The primers utilized on sequencing were the same as the second round PCR reaction. The sequences were edited on the DNAStar using both strand chromatograms.

### 3.4. Sequence Analysis

The sequences were aligned using ClustalW algorithm. The distances and the phylogeny were computed using Mega 5.1 and compared to reference sequences obtained from Los Alamos Laboratory HIV Sequence Database. The phylogenetic trees were computed using the Neighbor-Joining method using Kimura-2-parameters to evaluate the substitution rate and the bootstrap test were made (1000 replicas).

## 4. Conclusions

In this work we observed the same LTR sequence insertion found in Indian samples generating an additional NF-κB. Of note, most of the subtype C isolates from Brazil contains insertions duplicating RBEIII sites in LTR. Additionally, Brazilian isolates carrying LTR sequence insertions were found in the group of therapeutic failure (GII). However, our sample size is small, limiting the statistical significance of this association. A larger number of samples are required to examine the association unequivocally. The impact of the findings reported here needs to be evaluated in cell culture to measure the influence of different combinations of TFBS on viral replication.

## Supplementary Files

Supplementary File 1 Supplementary Materials (PDF, 338 KB)
