# Supplementary material for: Genetic Diversity in HIV-1 Subtype C LTR from Brazil and Mozambique Generates New Transcription Factor-Binding Sites
Source: Viruses. 2014 Jun 23;6(6):2495–504. doi: 10.3390/v6062495 (PMC4074939; doi:10.3390/v6062495)

Supplementary Materials

Genetic Diversity in HIV-1 Subtype C LTR from Brazil and Mozambique Generates New Transcription Factor-Binding Sites

José Boullosa, Mahesh Bachu, Dulce Bila, Udaykumar Ranga, Theodoro Süffert, Tomoko Sasazawa and Amilcar Tanuri

Suplementary Figure S1. Rooted neighbor-joining tree based on long terminal repeat (LTR) sequences of Mozambican HIV-1 isolates (marked with ●). HIV-1 subtype reference sequences were obtained from the GenBank databases and the accession numbers are shown in figure. The tree was rooted using an SIV sequence. Only bootstrap values >70% are shown.

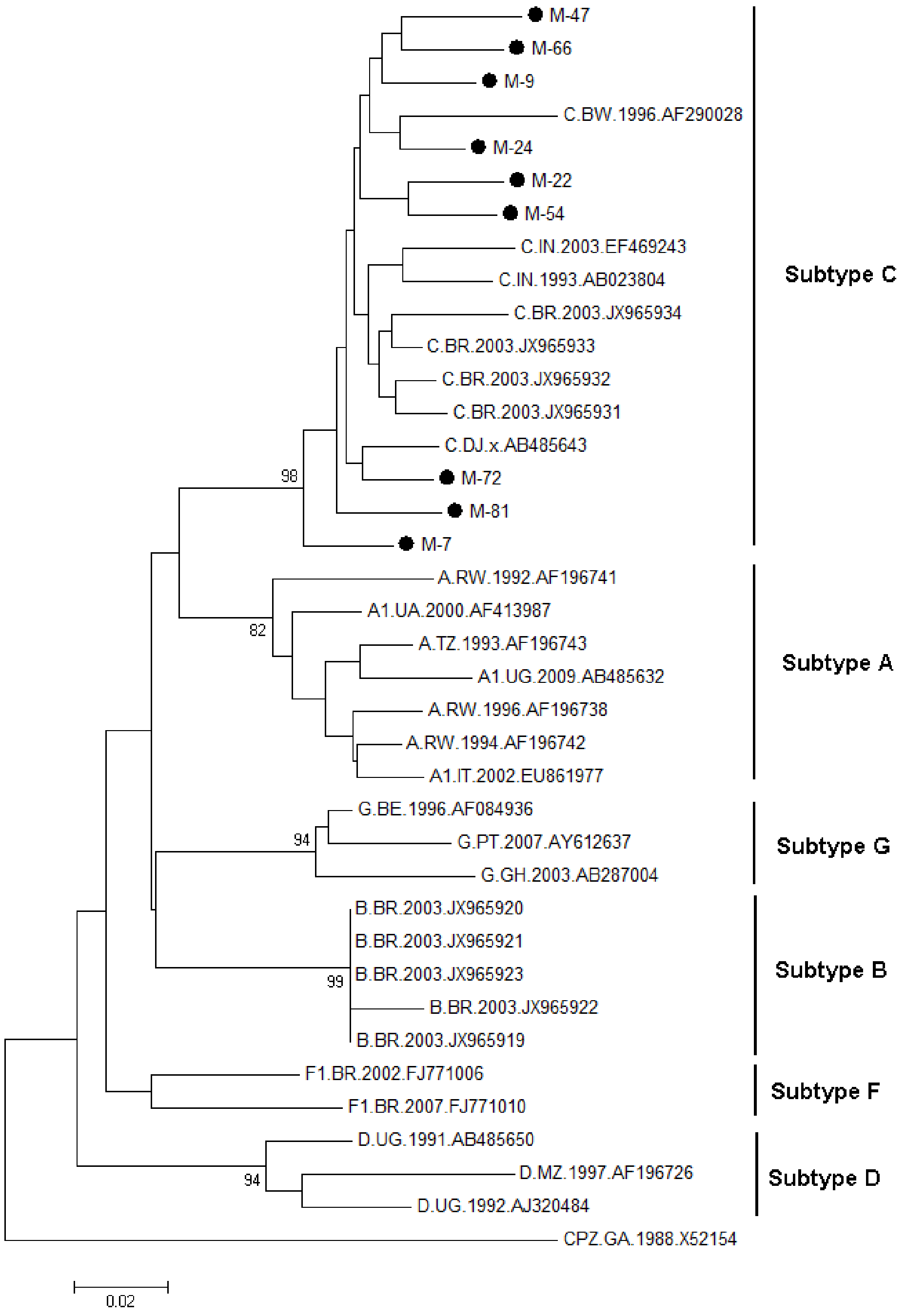

Suplementary Figure 2. Rooted neighbor-joining tree based on long terminal repeat (LTR) sequences of Brazilian HIV-1 isolates. The subtype C isolates is marked with ●, the subtype B isolate is marked with ■ and the subtype D isolate is marked with ♦. HIV-1 subtype reference sequences were obtained from the GenBank databases and the accession numbers are shown in figure. The tree was rooted using an SIV sequence. Only bootstrap values >70% are shown.

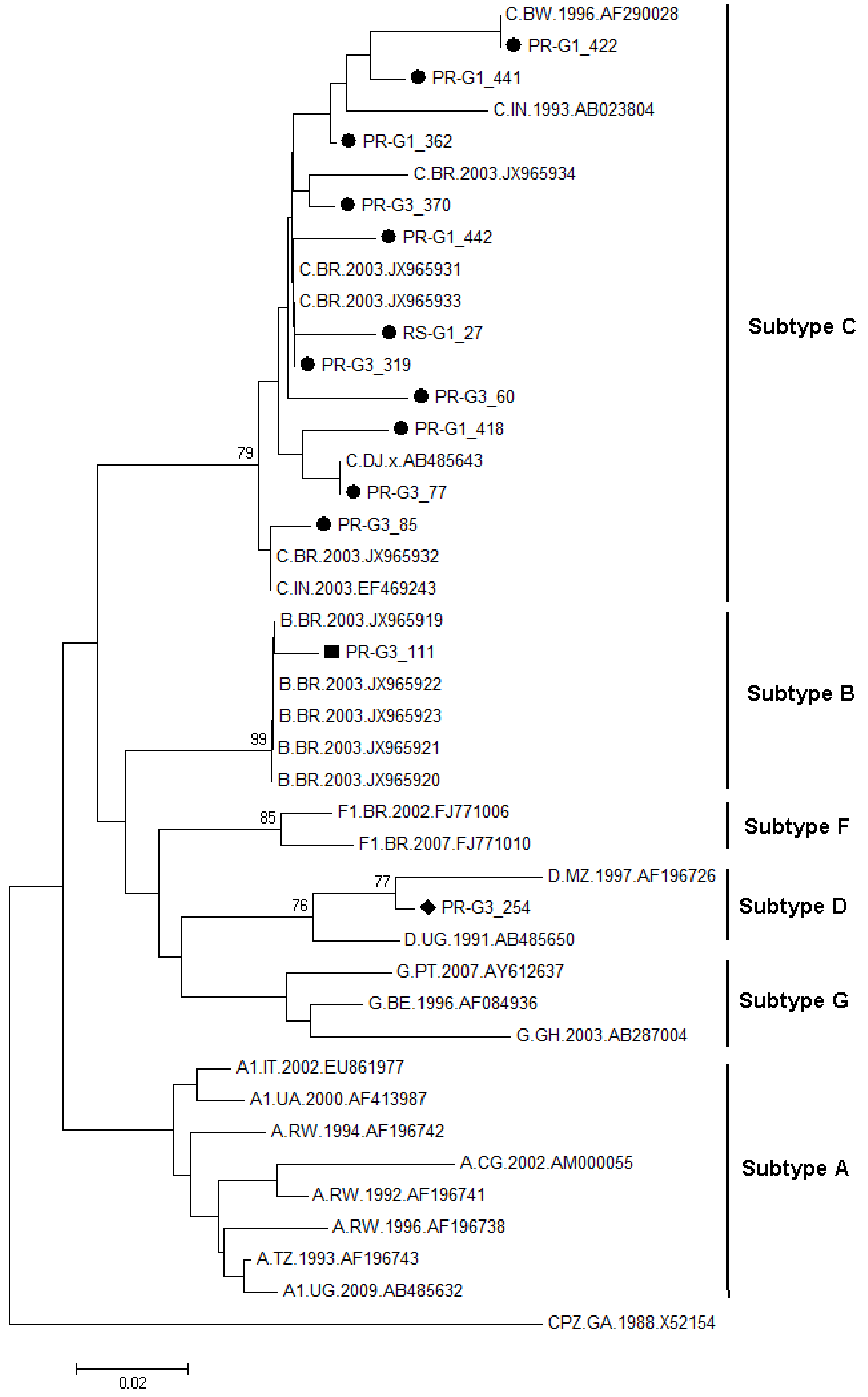

Suplementary Figure 3. Rooted neighbor-joining tree based on long terminal repeat (LTR) sequences of Brazilian HIV-1 isolates (marked with ●). HIV-1 subtype reference sequences were obtained from the GenBank databases and the accession numbers are shown in figure. The tree was rooted using an SIV sequence. Only bootstrap values >70% are shown.

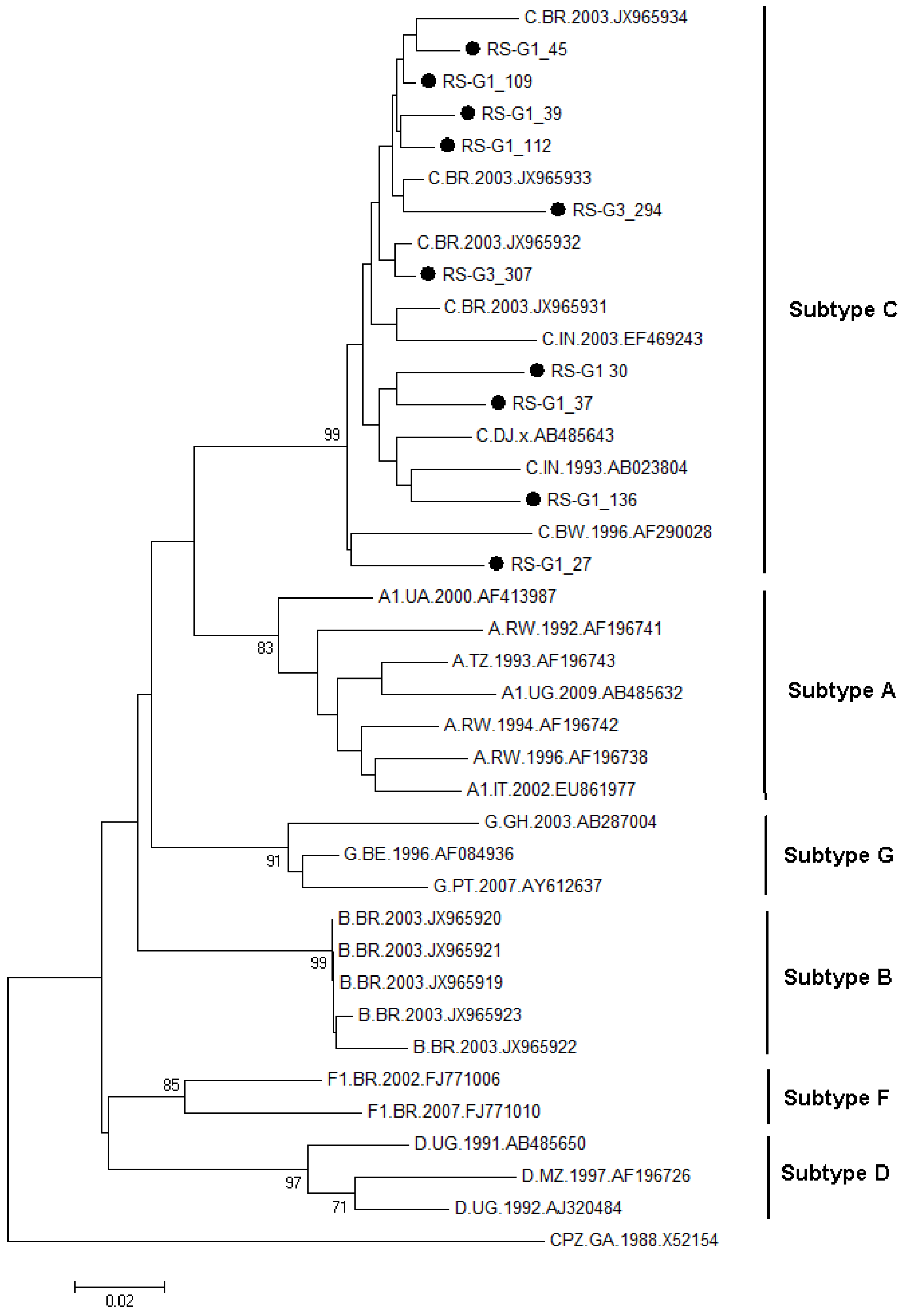

Supplement: Supplementary File 1 — Supplementary Materials (PDF, 338 KB) [file viruses-06-02495-s001.pdf]
